# Multifocal Inflammatory Myofibroblastic Tumor Presenting With Hemorrhagic Shock Due to Duodenal Invasion by a Pancreatic Head Mass: A Case Report

**DOI:** 10.7759/cureus.100483

**Published:** 2025-12-31

**Authors:** Issei Fukuda, Minh H Tran, Masaaki Akahane, Mina Komuta, Sakuzo W Honjo, Hajime Higuchi, Yuji Ohizumi, Kota Takahashi, Takuya Minagawa, Ryo Takagi, Mayu Yao, Taisei Katahira, Naoki Yoshioka, Shigeru Kiryu

**Affiliations:** 1 Radiology, International University of Health and Welfare - Narita Hospital, Narita, JPN; 2 Radiology, University of Tokyo Hospital, Tokyo, JPN; 3 Pathology, International University of Health and Welfare - Narita Hospital, Narita, JPN; 4 Oncology, International University of Health and Welfare - Narita Hospital, Narita, JPN; 5 Palliative Medicine, International University of Health and Welfare - Narita Hospital, Narita, JPN; 6 Hepato-Biliary-Pancreatic and Gastrointestinal Surgery, International University of Health and Welfare - Narita Hospital, Narita, JPN; 7 Otolaryngology, International University of Health and Welfare - Narita Hospital, Narita, JPN

**Keywords:** alk-negative, erbb2 protein, inflammatory myofibroblastic tumor (imt), transarterial embolization, upper gastrointestinal (ugi) bleeding

## Abstract

Inflammatory myofibroblastic tumor (IMT) is a rare mesenchymal neoplasm of intermediate malignant potential. Although typically indolent, IMT can occasionally exhibit aggressive behavior, including distant metastasis or multifocal involvement. Clinical manifestations vary widely according to anatomic location. Gastrointestinal tract involvement has been reported; however, massive gastrointestinal bleeding leading to hemorrhagic shock is exceedingly rare.

We present a 56-year-old man who presented with melena and hemorrhagic shock. Imaging demonstrated a pancreatic head mass with duodenal ulceration, along with a pulmonary nodule and multiple renal lesions. Endoscopic hemostasis was unsuccessful, and transcatheter arterial embolization (TAE) achieved temporary bleeding control. The patient subsequently underwent pancreaticoduodenectomy for definitive hemostasis and local disease management. Histopathologic examination of the pancreatic mass demonstrated spindle cell proliferation consistent with IMT and negative immunoreactivity for anaplastic lymphoma kinase (ALK), c-ros oncogene 1 (ROS1), and other relevant markers. Systemic evaluation revealed multifocal disease involving the brain, parotid gland, lungs, heart, liver, pancreatic tail, kidneys, adrenal gland, lymph nodes, and bones. Dynamic contrast-enhanced magnetic resonance imaging (MRI) of the parotid lesion showed gradual, continuous enhancement, consistent with a fibrous-rich lesion. Based on biopsies from multiple sites and the overall clinical course, a diagnosis of multifocal IMT was established. Initial systemic therapy with doxorubicin achieved stable disease at three months. Based on the genomic findings identified following comprehensive genomic profiling, the patient was subsequently enrolled in the DESTINY-PanTumor02 clinical trial and transitioned to treatment with trastuzumab deruxtecan targeting the human epidermal growth factor receptor 2 (HER2) alteration.

Our case represents an exceptionally rare presentation of IMT, characterized by life-threatening gastrointestinal bleeding and extensive multifocal dissemination. It illustrates the marked radiologic and clinicopathologic heterogeneity of IMT and emphasizes the importance of comprehensive histopathologic and molecular evaluation to guide management in aggressive disease variants. Close collaboration across cross-disciplinary diagnostic specialties is essential for the assessment and treatment of rare, multi-organ conditions.

## Introduction

Inflammatory myofibroblastic tumor (IMT) is a rare mesenchymal neoplasm, classified by the World Health Organization as an intermediate-grade malignancy, accounting for <1% of all soft tissue tumors [1]. IMT predominantly affects children and young adults but occurs across a wide age spectrum [2]. There is a slight female predominance; however, tumor location may impact both the age of onset and gender distribution [2]. Histologically, IMT is distinguished by spindle-shaped myofibroblastic cells accompanied by a dense inflammatory infiltrate, typically composed of plasma cells, lymphocytes, and eosinophils [1,2]. Although IMT predominantly affects children and young adults, it can arise across the entire age spectrum [1]. The lung, abdominal cavity, and retroperitoneum represent the most frequent primary sites of involvement [2]. 

Clinical presentation is nonspecific and primarily determined by tumor location. Systemic inflammatory features, including fever, malaise, weight loss, and anemia, occur in approximately 20%-30% of patients [2]. These findings often correlate with elevated nonspecific inflammatory markers, such as C-reactive protein, yet no disease-specific serum tumor markers have been identified. Gastrointestinal IMTs may lead to severe complications, such as obstruction or bleeding [1]. On imaging, IMTs typically appear as solitary, well-circumscribed soft tissue masses with heterogeneous enhancement on contrast-enhanced studies [2]. However, radiologic manifestations are variable, ranging from multiple lesions to irregular or ill-defined borders. In some cases, imaging features resemble malignant neoplasms, including sarcoma or carcinoma, necessitating histopathological examination through biopsy or surgical resection for definitive diagnosis [2,3].

IMTs are generally regarded as tumors with low metastatic potential, with reported rates <5%. However, local invasion and recurrence are not uncommon, and distant metastases to the lung, liver, brain, and bone have been described in rare cases [4]. Anaplastic lymphoma kinase (ALK) gene rearrangements are present in approximately 50%-60% of IMTs. Although some studies suggest a higher metastatic risk in ALK-negative tumors, other studies have found no significant association between ALK expression and metastatic behavior [5-7].

Regarding management, complete surgical resection remains the mainstay of treatment and is curative in the majority of localized cases; however, systemic therapy is necessary for disseminated or unresectable tumors [4]. Targeted therapy with ALK or c-ros oncogene 1 (ROS1) inhibitors, including crizotinib, has revolutionized the therapeutic landscape in fusion-positive cases [2].

We present an exceptionally rare case of IMT arising in the pancreatic head, resulting in duodenal ulceration and severe melena leading to hemorrhagic shock. Subsequent comprehensive evaluation demonstrated multifocal involvement of the brain, parotid gland, lung, heart, liver, kidney, lymph node, and bone. Our study emphasizes an uncommon radiologic presentation and highlights the aggressive potential of IMT, along with the associated diagnostic and therapeutic challenges.

## Case presentation

A 56-year-old man with a history of hypertension presented to the Emergency Department with three days of melena and new-onset dizziness on the day of admission. On arrival, he was alert and oriented; however, vital signs indicated tachycardia (heart rate, 150 beats per minute). Physical examination revealed a soft, non-tender, non-distended abdomen without notable abnormalities. Initial laboratory evaluation showed severe anemia, hypoalbuminemia, mildly elevated liver transaminase levels, a slight increase in C-reactive protein, and elevated lactate (Table 1).

**Table 1:** Laboratory data at initial evaluation Reference values vary by patient population and laboratory methodology. ^1^ Lactate was measured from venous whole blood using blood gas analysis, whereas all other laboratory parameters were obtained from serum or plasma. The approximate reference range for venous blood gas lactate is 0.5-2.2 mmol/L.

Parameter	On evaluation	Reference range (adults)
Hemoglobin (g/dL)	4.2	13.7-16.8
Hematocrit (%)	13.3	40.7-50.1
Platelet count (×103/μL)	246	158.0-348.0
White-cell count (×103/μL)	9.75	3.30-8.60
Prothrombin time (sec)	8.8	10.0-13.0
Activated partial thromboplastin time (sec)	19.2	25.0-40.0
Fibrinogen (mg/dL)	298	160.0-350.0
Albumin (g/dL)	2.3	4.1-5.1
Blood urea nitrogen (mg/dL)	27.9	8.0-20.0
Creatinine (mg/dL)	0.77	0.65-1.07
Total bilirubin (mg/dL)	0.4	0.4-1.5
C-reactive protein (mg/dL)	1.55	0.00-0.14
Aspartate aminotransferase (U/L)	80	13-30
Alanine aminotransferase (U/L)	65	10-42
Lactate dehydrogenase (U/L)	158	124-222
Amylase (U/L)	67	44-132
Carcinoembryonic antigen (ng/mL)	0.7	0.0-5.0
Carbohydrate antigen 19-9 (U/mL)	9	0.0-37.0
Span-1 (U/mL)	6.8	0-30
DUPAN-2 (U/mL)	33	0-150
Lactate (mmol/L)^1^	4.5	-

**Figure 1:**
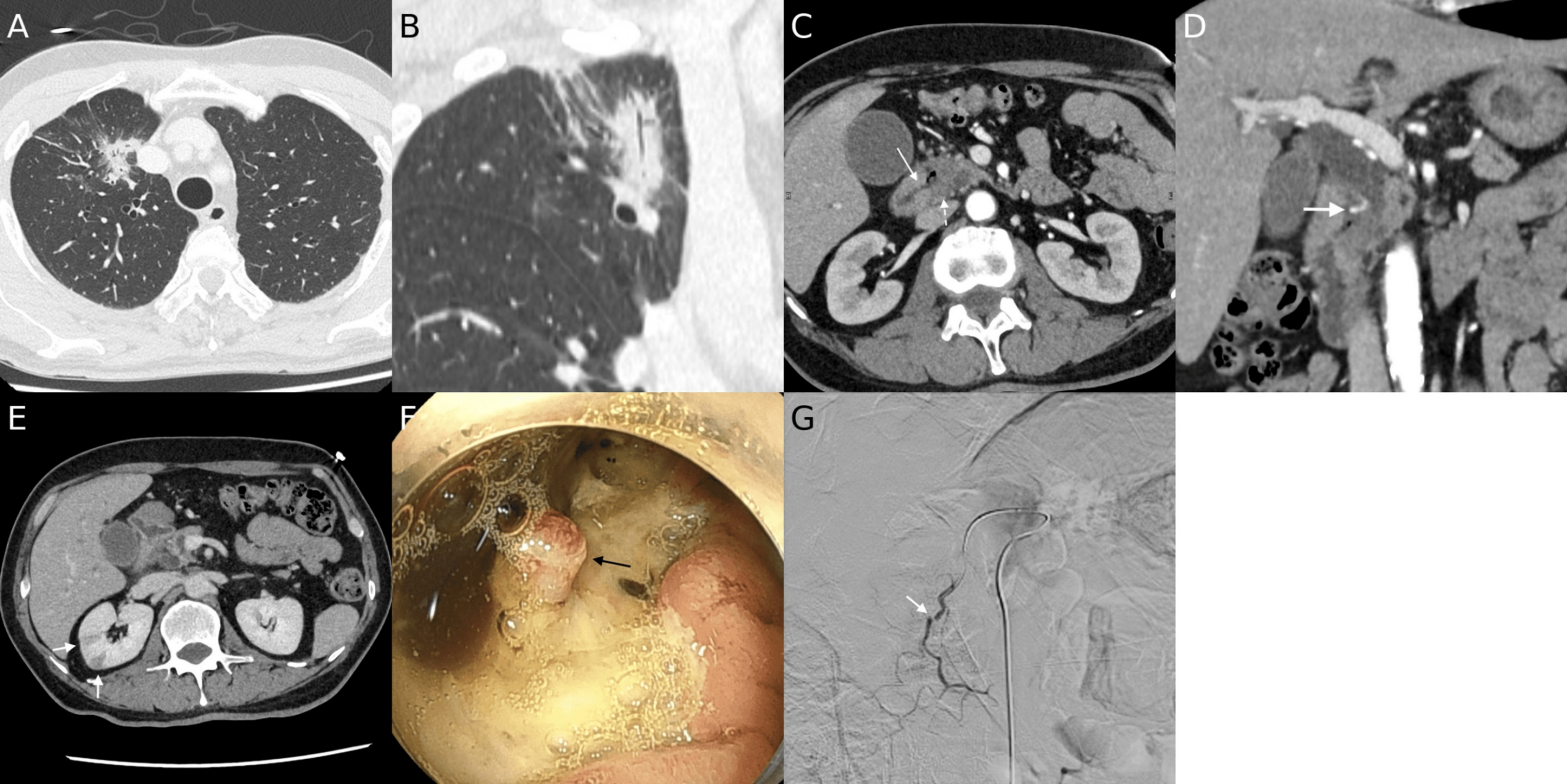
Initial contrast-enhanced computed tomography (CT) and angiographic findings at presentation (A, B) Right upper lobe pulmonary irregular consolidation, surrounded by ground-glass opacity and coarse spiculation, shown on axial (A) and coronal (B) images. (C, D) Hypovascular mass of the pancreatic head (dashed arrow). Axial image (C) demonstrates discontinuity of the duodenal wall (arrow), consistent with ulcerative invasion. Coronal image (D) shows biliary duct dilatation and a pseudoaneurysm of the posterior superior pancreaticoduodenal artery (PSPDA, arrow). (E) Wedge-shaped, hypoenhanced areas in the right kidney (arrow). (F) Upper gastroendoscopic image. A duodenal ulcer extending from the superior angle to the descending portion, with a 10-mm exposed artery at its center. No mass-forming lesion was identified. (G) Digital subtraction angiography (DSA) demonstrated disruption of the PSPDA at the location corresponding to the pseudoaneurysm previously identified on CT.

Contrast-enhanced computed tomography (CT) demonstrated a 27-mm irregular consolidation with surrounding ground-glass opacity and pleural indentation in the right upper lung lobe (Figures 1A, 1B). In addition, a 2-cm hypovascular mass was identified in the pancreatic head, accompanied by dilation of the biliary and pancreatic ducts and adjacent duodenal ulceration (Figures 1C, 1D). A pseudoaneurysm of the posterior superior pancreaticoduodenal artery (PSPDA) was suspected as the source of bleeding (Figure 1E). Multiple wedge-shaped, hypoenhanced areas were observed in both kidneys (Figure 1F). Upper gastrointestinal endoscopy revealed a duodenal ulcer extending from the superior angle to the descending portion, with a 10-mm exposed artery at its base, where hemostasis could not be achieved. No mass-like lesion was observed. Subsequent transarterial angiography demonstrated disruption of the PSPDA (Figure 1G). The guidewire extravasated into the ulcer crater, confirming arterial perforation. Transcatheter arterial embolization (TAE) with coils and N-butyl cyanoacrylate successfully achieved hemostasis.

Following stabilization of gastrointestinal bleeding, endoscopic ultrasound-guided tissue acquisition was performed to evaluate the pancreatic lesion. Histopathology demonstrated spindle cell proliferation without definitive malignant features. Immunohistochemistry (IHC) showed weak positivity for α-smooth muscle actin and negativity for ALK, gastrointestinal stromal tumor protein 1 (DOG1), CD117 (c-kit), CD34, S100 protein, signal transducer and activator of transcription 6 (STAT6), and β-catenin. These findings were suggestive of IMT; however, reactive changes secondary to recent embolization could not be excluded. Additional evaluation of the right upper lobe lesion included sputum cultures and tumor markers, including pro-gastrin-releasing peptide, carcinoembryonic antigen, and cytokeratin-19 (CK19) fragment, all of which were negative.

Two weeks later, the patient returned to the Emergency Department with recurrent melena and dizziness, requiring further blood transfusion. Imaging findings were similar to those from the prior admission; however, progressive local tumor invasion was suspected to have eroded into the gastroduodenal artery (GDA), resulting in active hemorrhage. Emergent TAE was again performed.

**Figure 2:**
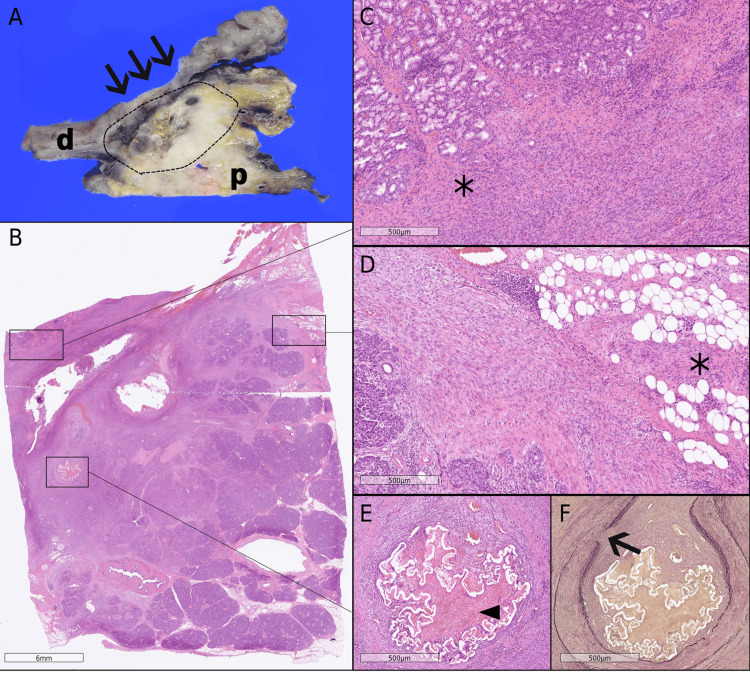
Gross and microscopic features of the pancreatic tumor (A) Gross specimen from pancreaticoduodenectomy. Duodenum (d) and pancreatic head (p) with an ill-circumscribed, gray-white tumor (outlined by dotted lines) and duodenal ulceration (arrows). (B) Low-power view of the tumor (marked by boxes), hematoxylin and eosin (H&E), scale bar = 6 mm. (C, D) High-power views demonstrating tumor (*) infiltrating the duodenum and peripancreatic fat, H&E, scale bar = 500 µm. (E) Tumor invasion into the arterial wall; arrowhead indicates preoperative embolization material, H&E, scale bar = 500 µm. (F) Elastica van Gieson stain showing tumor infiltration of the arterial wall (arrow), scale bar = 500 µm.

**Figure 3:**
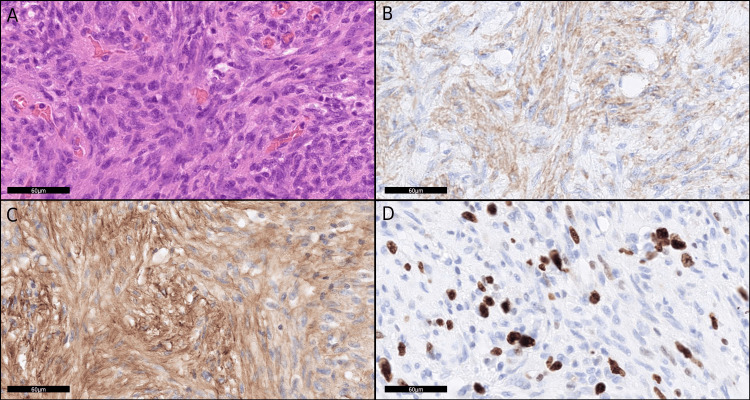
Immunohistochemical features of the tumor (A) Spindle-cell proliferation with lymphoplasmacytic infiltration; no nuclear atypia or mitotic activity observed, hematoxylin and eosin (H&E), scale bar = 60 µm. (B) Tumor cells positive for alpha-smooth muscle actin (αSMA), scale bar = 60 µm. (C) Tumor cells positive for fibronectin, scale bar = 60 µm. (D) Ki-67 proliferation index of approximately 30%, scale bar = 60 µm.

Due to the ongoing risk of life-threatening hemorrhage and concern for an underlying neoplasm, the patient underwent pylorus-preserving pancreaticoduodenectomy. Gross examination revealed an ill-circumscribed, solid, gray-white mass measuring 50 × 28 mm, with an associated ulcerative lesion in the duodenum (Figure 2A). The tumor exhibited an infiltrative growth pattern, extending into the duodenum, peripancreatic fat, lymph nodes, and bile duct wall (Figures 2B-2D). Obliterative vasculitis and lymphoid follicle formation were present, consistent with changes related to preoperative embolization (Figure 2E). Elastica van Gieson staining demonstrated tumor infiltration into the arterial wall (Figure 2F). On high-power microscopy, the lesion consisted of densely packed spindle cells arranged in fascicular and storiform patterns, accompanied by lymphoplasmacytic infiltration and scattered eosinophils. No significant nuclear atypia, mitotic activity, or necrosis was observed (Figure 3A). IHC demonstrated focal positivity for α-smooth muscle actin (Figure 3B) and fibronectin (Figure 3C). Tumor cells were negative for desmin, myogenin, human melanoma black 45 (HMB45), MelanA, CD31, CD34, c-kit, ERG, DOG1, STAT6, S100, cytokeratin (AE1/AE3), cytokeratin 5/6 (CK 5/6), CD45 (leukocyte common antigen), CD68, CD21, CD23, β-catenin, ALK, and mouse double minute 2 homolog (MDM2). Small clusters of IgG4-positive cells were observed, with a peak count of 24 cells per high-power field in hot spots. Ki-67 immunostaining demonstrated an approximately 30% proliferation index (Figure 3D). Taken together, the morphological and immunohistochemical findings confirmed the diagnosis of IMT.

**Figure 4:**
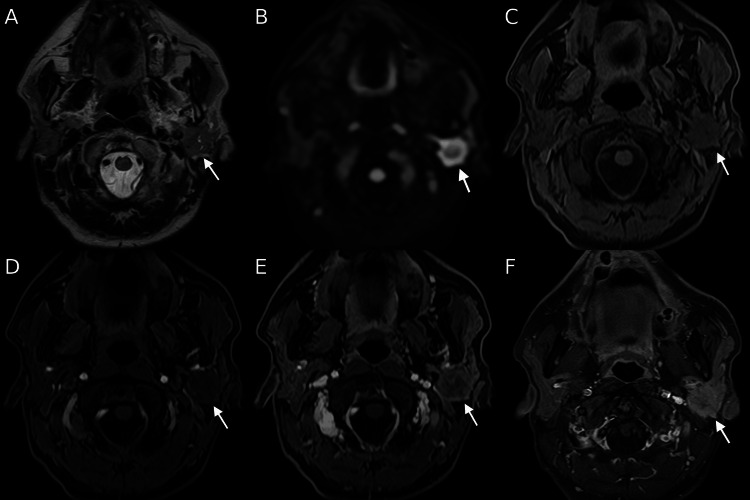
Magnetic resonance imaging (MRI) of the left parotid lesion The lesion (arrow) appeared slightly hyperintense on T2-weighted imaging (A) and hyperintense on diffusion-weighted imaging, with an apparent diffusion coefficient of approximately 0.9 × 10⁻³ mm²/s (B). On dynamic contrast-enhanced sequences, the lesion (arrow) demonstrated mild, progressive enhancement in the early phase and relatively marked enhancement in the delayed phase (C-F). Percutaneous biopsy confirmed inflammatory myofibroblastic tumor.

Around the same period, the patient developed progressive left facial nerve palsy. CT of the head revealed a lesion in the left parotid gland. Magnetic resonance imaging (MRI) demonstrated a hypointense signal on fat-saturated T1-weighted images, slight hyperintensity on T2-weighted images, and hyperintensity on diffusion-weighted images, with an apparent diffusion coefficient of approximately 0.9 × 10⁻³ mm²/s (Figures 4A-4C). Dynamic contrast-enhanced imaging exhibited mild, progressive enhancement in the early phase and relatively marked enhancement in the delayed phase, a pattern suggestive of a fibrous-rich lesion (Figures 4D-4F).

**Figure 5:**
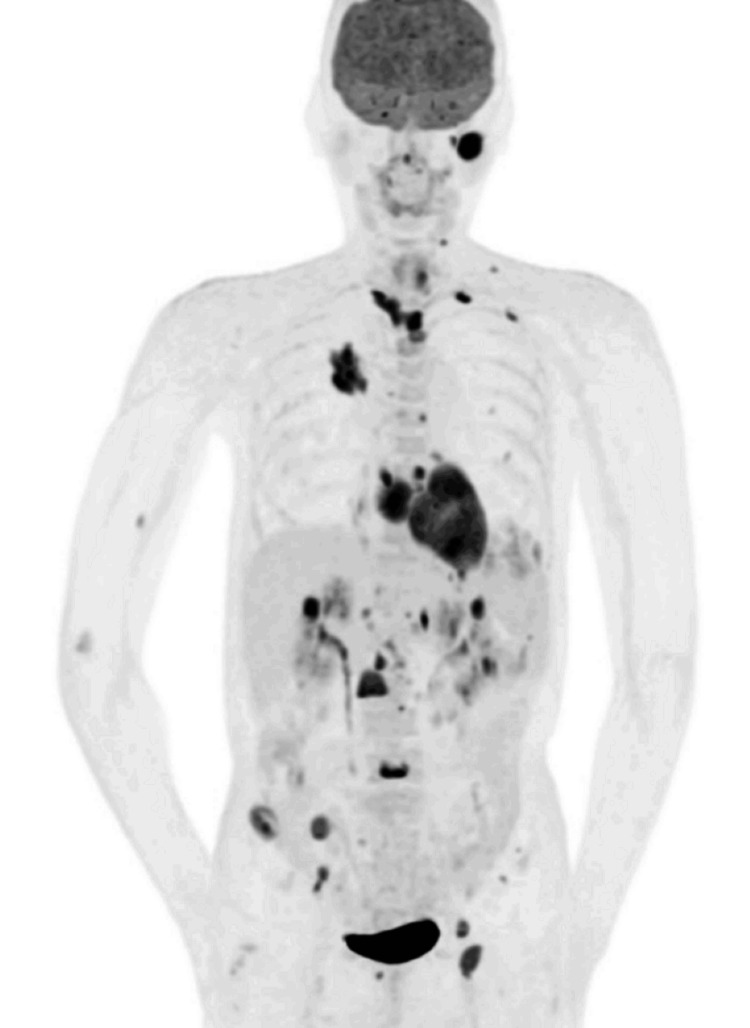
Maximum-intensity projection (MIP) image from ¹⁸F-fluorodeoxyglucose positron emission tomography/computed tomography (¹⁸F-FDG PET/CT) The figure demonstrates multifocal FDG-avid lesions of the brain, left parotid gland, lungs, heart, liver, pancreatic tail, kidneys, left adrenal gland, lymph nodes, and bones.

**Figure 6:**
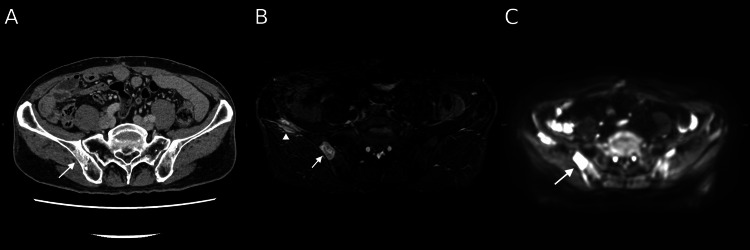
Multimodality imaging of the right iliac bone lesions Computed tomography exhibits ill-defined areas of increased medullary attenuation (A). Magnetic resonance imaging demonstrates slightly heterogeneous hyperintensity on short tau inversion recovery (STIR) sequence (B) and hyperintensity on diffusion-weighted imaging (C), with an apparent diffusion coefficient of approximately 1.2 × 10⁻³ mm²/s. One lesion extends into adjacent soft tissue, with preservation of the cortical bone (arrowhead). Percutaneous biopsy of one lesion (arrow) confirmed inflammatory myofibroblastic tumor.

Further systemic evaluation with contrast-enhanced CT, MRI, and ^18^F-fluorodeoxyglucose positron emission tomography (PET)/CT demonstrated multiple lesions involving the brain, left parotid gland, lungs, heart, liver, pancreatic tail, kidneys, left adrenal gland, lymph nodes, and bones (Figure 5). Some lesions newly appeared within a short interval, whereas others exhibited relatively rapid enlargement on serial imaging. Osseous lesions were characterized by ill-defined areas of increased medullary attenuation on CT, with several demonstrating extension into adjacent soft tissues while preserving cortical integrity on MRI (Figures 6A-6C).

**Figure 7:**
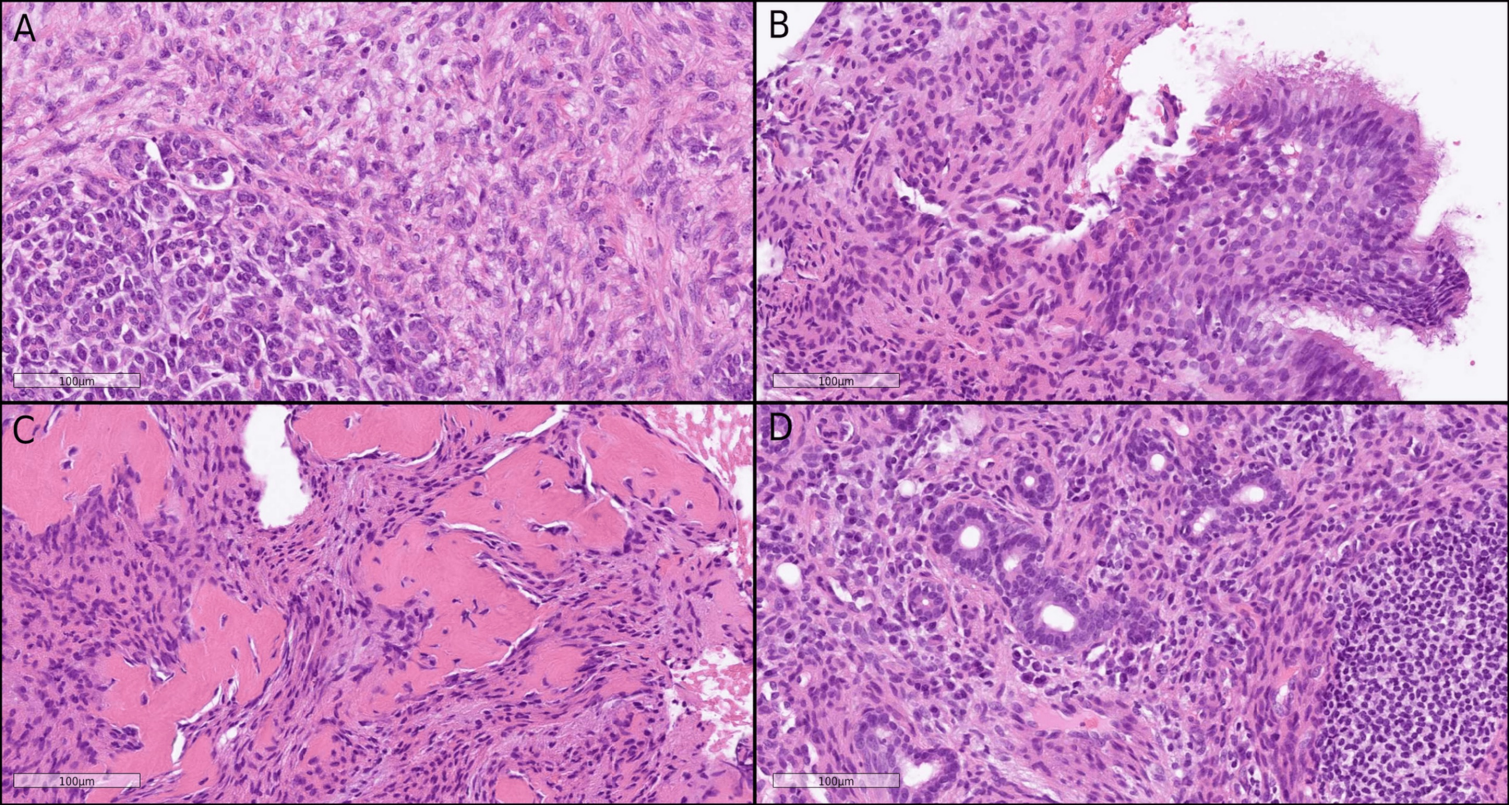
Multifocal tumor involvement (A) Tumor in the resected pancreatic head, hematoxylin and eosin (H&E), scale bar = 100 µm. (B) Tumor in lung biopsy, H&E, scale bar = 100 µm. (C) Tumor in bone marrow biopsy, H&E, scale bar = 100 µm. (D) Tumor in parotid gland biopsy, H&E, scale bar = 100 µm.

Similar to the pancreatic head tumor, transbronchial lung biopsy of the right upper lobe demonstrated spindle cell proliferation without evidence of primary lung carcinoma (Figures 7A, 7B). Considering the imaging findings and clinical context, additional immunohistochemical analyses were performed and exhibited a profile consistent with the pancreatic lesion, raising suspicion for multifocal IMT. Percutaneous biopsies of the right iliac bone and left parotid gland confirmed histopathological and immunohistochemical features identical to those of the pancreatic head tumor (Figures 7C, 7D). Based on the pathological findings from the resected pancreatic specimen and biopsied lung, parotid, and bone lesions, along with interval progression of disease on imaging, the extensive systemic lesions were ultimately diagnosed as multifocal IMT.

Following a multidisciplinary team conference to discuss therapeutic options, systemic therapy was initiated, considering the infeasibility of complete surgical resection. In view of the high likelihood of actionable genetic alterations, a comprehensive genomic panel was submitted. Meanwhile, due to the aggressive clinical course, which necessitated immediate intervention prior to the availability of genomic results, single-agent doxorubicin was selected as the initial treatment, following the standard regimen for high-grade soft-tissue sarcoma. Comprehensive genomic profiling was performed on tumor tissue from the pancreatic head, the suspected primary lesion, using the NGS-based FoundationOne® CDx assay (Foundation Medicine, Inc., Cambridge, MA, USA), analyzing 324 cancer-related genes, which demonstrated ERBB2 amplification (copy number 11). Additionally, IHC revealed weak human epidermal growth factor receptor 2 (HER2) expression, whereas HER2 dual in situ hybridization confirmed gene amplification. Throughout the clinical course, we found no laboratory markers predictive of disease activity. Consequently, the assessment of treatment response and disease progression was primarily conducted through cross-sectional imaging and clinical evaluation. At the three-month follow-up, contrast-enhanced CT showed stable disease. Given the identification of ERBB2 amplification and the potential for targeted therapy, the patient was subsequently enrolled in the DESTINY-PanTumor02 clinical trial (NCT04482348) and initiated treatment with trastuzumab deruxtecan.

## Discussion

We describe an exceptionally rare case of IMT presenting with hemorrhagic shock due to upper gastrointestinal bleeding from duodenal invasion by a pancreatic head mass. Subsequent evaluation demonstrated multifocal disease. Although the pulmonary lesion was initially suspected to represent an unrelated malignancy, correlation of histopathologic findings from the pancreatic and additional biopsy specimens, together with imaging evidence of multifocal lesions, confirmed systemic dissemination of IMT. Our study emphasizes the importance of multidisciplinary diagnostic collaboration when rare diseases involve multiple organs, as coordinated interpretation across specialties is critical for developing a coherent and accurate differential diagnosis.

Upper gastrointestinal bleeding as the initial manifestation of IMT is exceedingly rare. To our knowledge, no prior case has reported IMT causing massive hemorrhage leading to hemorrhagic shock [8,9]. In most documented cases, bleeding originates directly from the tumor mass itself [8]. In contrast, our patient developed duodenal ulceration from tumor invasion, which subsequently eroded major vessels, including the PSPDA and GDA. The absence of an intraluminal tumor on endoscopy, together with angiographic evidence of guidewire extrusion into the duodenal lumen, strongly supported vessel rupture secondary to tumor infiltration. Elastica van Gieson staining confirmed arterial wall disruption caused by tumor invasion; this mechanism represents an exceptionally rare clinical presentation of IMT.

Retrospectively, the imaging features of each lesion were atypical and nonspecific. The pancreatic lesion was ill-defined and demonstrated progressive, weak contrast enhancement, a pattern potentially compatible with IMT but also consistent with pancreatic carcinoma. The pulmonary lesion was spiculated, with an associated pleural tag, and lacked a distinct mass, which was atypical for IMT and more suggestive of carcinoma. IMT involving the parotid gland is rarely reported [10]. Although the gradually increasing enhancement, with relatively strong delayed-phase uptake, in the parotid lesion resembled previously described IMT characteristics in other organs, such as the spleen, the MRI findings were nondiagnostic and did not exclude carcinoma [3]. In addition, infiltrative extension with partial preservation of anatomical structures, observed in the pancreatic and bone lesions, is uncommon; however, a few comparable cases have been documented [11,12].

Our case was negative for ALK rearrangement by IHC and molecular testing. ALK rearrangements are identified in approximately 50%-60% of IMTs; however, the relationship between ALK status and tumor aggressiveness remains uncertain [5-7]. Notably, genomic profiling in our patient revealed ERBB2 amplification, with weak HER2 expression on IHC. To our knowledge, although HER2 expression and amplification have been rarely reported in select sarcomas, they have not been previously documented in IMT. IMT harbors diverse genetic alterations, including ALK, ROS1, rearranged during transfection (RET), and neurotrophic tropomyosin receptor kinase rearrangements [4,13]. Considering the marked clinical and radiologic heterogeneity of IMT, current IMT classifications may ultimately be refined into distinct molecular subsets, consistent with prior studies [13]. Comprehensive genomic testing should be considered in ALK-negative cases, as well as in aggressive or refractory disease, to identify novel molecular drivers and support emerging targeted therapeutic strategies while clarifying genotype-phenotype correlations. 

Treatment of multifocal IMT is challenging. Surgical resection remains the primary approach for localized disease; however, systemic therapy is necessary for disseminated or unresectable tumors [4,8,14]. Due to the rarity of advanced IMT, no standardized chemotherapy regimen exists, and management is often extrapolated from protocols for high-grade soft tissue sarcoma, with doxorubicin considered a reasonable first-line option [15]. Retrospective studies have reported response rates of approximately 50% with anthracycline-based regimens, such as doxorubicin, which exceed those typically observed in other soft tissue sarcomas [16]. Targeted therapy with ALK or ROS1 inhibitors, including crizotinib, has demonstrated substantial activity in fusion-positive IMT, yielding durable responses; however, resistance can occur [17,18]. Considering the ALK-negative status, aggressive clinical behavior, and high tumor burden in our case, systemic therapy with doxorubicin was initiated promptly while awaiting genomic analysis.

## Conclusions

We present an exceptionally rare case of IMT presenting with gastrointestinal bleeding and multifocal dissemination. Our study emphasizes the significant clinical and radiological heterogeneity of IMT and underscores the pivotal role of multidisciplinary diagnostic collaboration in ensuring a coherent and thorough evaluation. Comprehensive genomic profiling in atypical or aggressive cases, coupled with continued studies, may yield important insights and inform future therapeutic strategies.
